# Bacterial and clinical metabolic signatures and their interactions in obese patients post-bariatric surgery

**DOI:** 10.1186/s12876-024-03450-1

**Published:** 2024-10-12

**Authors:** Mengjie Hu, Qiaoyuan Xiang, Zixuan Mei, Cheng Gong, Dingyu Pan, Yumin liu, Zhen Li

**Affiliations:** 1 Department of Hepatobiliary & Pancreatic Surgery, Zhongnan Hospital of Wuhan University, Wuhan, 430071 PR China; 2 Bariatric and Metabolic Diseases Surgery Center, Zhongnan Hospital of Wuhan University, Wuhan, 430071 PR China; 3 Neurology Department, Zhongnan Hospital of Wuhan University, Wuhan, 430071 PR China

**Keywords:** Laparoscopic Sleeve Gastrectomy, Gut microbiota, Obesity, 16S rRNA analysis

## Abstract

**Background:**

Obesity is a growing health concern in China, closely linked to metabolic disorders such as type 2 diabetes. Laparoscopic Sleeve Gastrectomy (LSG) is effective in promoting weight loss and improving metabolic outcomes. Emerging evidence highlights the role of gut microbiota in metabolic regulation, yet the specific alterations in gut microbiota and their association with metabolic changes post-surgery in Chinese patients remain unclear. Understanding these shifts could provide key insights into optimizing treatment strategies for metabolic improvement following bariatric surgery.

**Methods:**

Stool samples and clinical data were collected from 30 obese patients before and 6 months after surgery. The composition of the gut microbiota was analyzed through 16S rRNA sequencing, and Spearman correlation analysis was used to determine the association between gut microbiota and clinical indicators.

**Results:**

The analysis of 30 patients showed a significant decrease in Body Mass Index (BMI) (36.75 ± 4.09 kg/m^2^ vs 26.37 ± 3.47 kg/m^2^, *p* < 0.0001). Glucose metabolism, including Hemoglobin A1C levels, improved significantly (6.05 ± 0.96 vs 5.05 ± 0.25, *p* < 0.0001), and liver function as well as serum lipid levels were also notably improved. LSG increased the richness and composition of gut microbiota in obese patients post-surgery. These changes in gut microbiota were closely associated with improved clinical metabolic parameters.

**Conclusion:**

LSG not only significantly reduces body weight while also alleviating metabolic syndrome and comorbidities by altering gut microbiota.

**Supplementary Information:**

The online version contains supplementary material available at 10.1186/s12876-024-03450-1.

## Introduction

Over the past 40 years, rapid economic growth, globalization, and urbanization,have led to a sharp rise in overweight and obesity rates in China. Among children and adolescents aged 7-18, the prevalence of overweight and obesity increased from 1% and 0.1% in 1985 to 14.0% and 6.4% in 2014, respectively. Similarly, the rates among Chinese adults have surged [1]. Recent national surveys indicate that over half of Chinese adults are now classified as overweight or obese based on Chinese population standards [2]. Overweight and obesity are major risk factors for cardiovascular diseases [3], type 2 diabetes [4], and certain cancers [5]. Bariatric surgery is the preferred treatment for severe obesity, offering improvements in obesity-related complications, quality of life, and overall mortality reduction [6]. Laparoscopic sleeve gastrectomy (LSG) is the most common bariatric surgery, comprising 60% of all weight loss procedures [7, 8]. Long-term randomized controlled trials (RCTs) have demonstrated that LSG, due to its simplicity, significant weight loss, and fewer postoperative complications, has become the mainstream approach for weight reduction [9].

Research suggests a bidirectional relationship between obesity and gut microbiota [10]. Disruption of gut microbiota in obese patients contributes to insulin resistance, chronic inflammation, and metabolic disorders [11],but the underlying mechanisms remain unclear. Additionally, obese individuals often experience deficiencies in vitamins and minerals, affecting the synthesis of trace elements by the gut microbiota [12, 13]. However, studies on the effects of bariatric surgery on gut microbiota have yielded inconsistent results. For instance, Zhang et al. reported a decrease in Firmicutes in obese patients in the United States post-surgery [14], while Campisciano et al. found a reduction in Bacteroidetes in Italian patients [15], and Pajecki et al. observed a decrease in Proteobacteria abundance in Spanish patients, with no changes in Firmicutes and Bacteroidetes [16]. Conversely, Kural et al. noted an increase in the Firmicutes-to-Bacteroidetes ratio in Turkish patients after surgery [17]. These discrepancies highlight the influence of ethnicity and geographical location on gut microbiota composition and diversity [18]. Therefore, it is essential to investigate gut microbiota changes in different populations and regions and to explore its role in metabolic disorders.

In this study, we collected fecal samples and clinical data from 30 patients before and after LSG surgery. Then we performed 16S rRNA sequencing to analyze changes in gut microbiota composition, and employed spearman analysis aimed to elucidate how the gut microbiota influence host metabolism.

## Data and methods

### Inclusion and exclusion criteria

Fecal samples were collected from patients undergoing Laparoscopic Sleeve Gastrectomy (LSG) at Zhongnan Hospital of Wuhan University. Eligible patients were aged 18-65 years and met the obesity surgery treatment guidelines [19]. Demographic and clinical data, including age, gender, weight, height, and admission examination results, were recorded. The exclusion criteria were: (1) history or presence of neurological or severe psychiatric disorders; (2) confirmed secondary or drug-induced obesity, or genetically determined obesity; (3) pregnancy or lactation; (4) inability to cooperate with the examinations; (5) use of antibiotics, probiotics, or prebiotics within the previous three months or during the study period; (6) severe primary diseases; (7) severe complications or safety issues; (8) voluntary withdrawal or refusal to continue participation.

All samples and clinical data were collected with informed consent, and the study was approved by the Ethics Review Committee of Zhongnan Hospital of Wuhan University (No. 2021005), in compliance with the Helsinki Declaration. Participants provided written consent after receiving detailed explanations prior to sample collection.

### Sample collection

Fecal and serum samples were collected from all patients one week before surgery and again six months postoperatively. Serological samples were analyzed within the hospital for laboratory parameters. For fecal sample collection, patients were instructed to obtain mid-segment stool samples using collection tubes. Samples were transported to the lab on dry ice, divided into two aliquots, and stored in 2 mL cryovials. All samples, after collection, were placed in liquid nitrogen for 15 min, then transferred to -80 °C for storage until further analysis.

### Preoperative preparation

All patients underwent a comprehensive preoperative evaluation by the Obesity and Metabolic Disease Surgery Center team, which included specialists from Obesity and Metabolic Disease Surgery, Endocrinology, Cardiology, Respiratory Medicine, Anesthesiology, Intensive Care, and Sleep Medicine. Multidisciplinary assessments were conducted, including cardiopulmonary function improvement exercises, to ensure thorough preparation and exclude any surgical contraindications.

### Surgical procedure

All surgeries were performed by the same experienced team at Zhongnan Hospital of Wuhan University, adhering to key guidelines [20]. A brief overview of the procedure is as follows: The patient was positioned supine with the head elevated and feet lowered, using a conventional 4-port approach. The greater curvature of the stomach was gradually dissected from the pylorus to the cardia, including the gastric colic and gastrosplenic ligaments, while the left gastric artery and short gastric arteries were ligated and divided. Dissection continued along the left side of the esophagus. A 36F gastric tube was inserted along the lesser curvature of the stomach. Approximately 4 cm above the pylorus, the greater curvature and gastric fundus were resected with a stapler up to the left side of the esophagus. The His angle was secured with a seromuscular stitch, and the incision line was reinforced with continuous seromuscular sutures, with optional omental reinforcement.

### Postoperative management

After surgery, all patients received treatment, including anti-infection measures, acid suppression, fluid replacement, and anticoagulation. Oral intake began 48 h postoperatively, and upper gastrointestinal iodine contrast imaging was performed on the third day. Upon confirming no gastric leakage and good gastrointestinal patency, patients were discharged.

Postoperative dietary guidelines, supervised by a case manager, were as follows: In the first week, patients consumed clear liquids. In weeks two to three, a full-liquid diet was recommended. From weeks four to six, a semi-liquid diet was introduced, followed by a transition to a low-calorie, balanced diet in weeks seven to twelve, eventually leading to a regular diet.

### Data collection

We reviewed inpatient and outpatient electronic medical records and collected fecal specimens both preoperatively and at 6 months postoperatively. Additionally, the following clinical data were collected:

1. Anthropometric measurements: Weight, BMI, waist circumference (WC), and hip circumference (HC).
2. Glucose metabolism indicators: Fasting plasma glucose (FPG), Hemoglobin A1C (HbA1c), and fasting insulin (FINS).
3. Lipid metabolism and biochemical markers: Total cholesterol (TC), low-density lipoprotein cholesterol (LDL-C), high-density lipoprotein cholesterol (HDL-C), triglycerides (TG), alanine aminotransferase (ALT), aspartate aminotransferase (AST), γ-glutamyl transferase (γ-GGT), and uric acid (UA).
4. FibroScan results: Liver stiffness measurement (LSM) and controlled attenuation parameter (CAP).

### 16S rRNA sequencing and statistical analysis

Fecal sample genomic DNA was isolated using the MagPure Stool DNA Kit (MAGEN, Shanghai, China), adhering to the manufacturer’s guidelines. And the purification of PCR products was conducted using AMpure XP magnetic beads (Beckman Coulter Genomics, Danvers, MA, USA). To avoid contamination, blank controls were applied during both DNA extraction and library setup, utilizing nucleic acid-free materials. Sample quality was assessed using an Agilent 2100 Bioanalyzer (Agilent Technologies, Santa Clara, CA, USA) and sequencing was performed on the Illumina HiSeq platform (Illumina, CA, USA).

Illumina sequencing was used to produce paired-end reads. FLASH software (v1.2.11) merged the reads according to overlap regions to generate specific tags. Trimmomatic software (v0.33) was used to discard low-quality tags, yielding around 75,000 clean tags per sample. By referencing the gold database (v20110519), chimeric sequences were identified and excluded. Tags with more than 97% similarity were clustered into OTUs (Operational Taxonomic Units) using USEARCH (v7.0.1090). Further chimera filtering was conducted with UCHIME software (v4.2.40) against the gold database. Species were annotated by comparing the sequences to the Silva database. The ggplot package in R (v3.4.1) was used to calculate beta diversity (the variation between samples). LEfSE (Linear Discriminant Analysis Effect Size) at the genus level created cladograms, and LDA (Linear Discriminant Analysis) revealed significant microbial shifts before and after surgery [21]. Continuous variables were expressed as mean ± standard deviation, and paired *t*-tests were performed to assess changes pre- and post-surgery. Spearman correlation analysis was conducted to examine the association between gut microbiota after bariatric surgery and key clinical indicators. Statistical significance was set at *p* < 0.05.

## Results

### lsg-induced weight loss and improved metabolic measures

Complete data from 30 obese patients (11 males, 19 females,) who underwent LSG were collected preoperatively and at 6 months postoperatively. The mean age was 34.03 ± 6.83 years, and the mean BMI was 36.75 ± 4.09 kg/m^2^. LSG was successfully performed on all patients, with no serious postoperative complications such as bleeding or gastric leaks reported within 6 months.

At 6 months postoperatively, patients showed substantial improvements in BMI (36.75 ± 4.09 vs 26.37 ± 3.47, *p* < 0.0001), waist circumference (WC) (115.95 ± 12.41 vs 91.81 ± 11.18, *p* < 0.0001), and hip circumference (HC) (117.15 ± 8.55 vs 100.90 ± 9.87, *p* < 0.0001). Metabolic measures also improved significantly, including fasting plasma glucose (FPG) (5.83 ± 1.55 vs 5.17 ± 0.34, *p* < 0.005), fasting C-peptide (FCP) (3.76 ± 1.09 vs 2.21 ± 0.64, *p* < 0.0001), and hemoglobin A1c (HbA1c) (6.05 ± 0.96 vs 5.05 ± 0.25, *p* < 0.0001). Blood lipid levels showed notable improvements, including total cholesterol (TC) (4.91 ± 0.86 vs 4.61 ± 0.85, *p < *0.05) 5.8 ± 1.55 vs 5.17 ± 0.34, *p* < 0.005), triglycerides (TG) (2.09 ± 1.15 vs 1.11 ± 0.39, *p < *0.0001) (3.76 ± 1.09 vs 2.21 ± 0.64, *p* < 0.0001), and high-density lipoprotein cholesterol (HDL-C) (1.12 ± 0.23 vs 1.29 ± 0.27, *p < *0.0001) (6.05 ± 0.96 vs 5.05 ± 0.25, *p* < 0.0001). Fatty liver measures, such as liver stiffness measurement (LSM) (10.03 ± 3.98 vs 6.83 ± 2.95, *p* < 0.001) and controlled attenuation parameter (CAP) (330.21 ± 22.73 vs 258.17 ± 27.47, *p* < 0.0001), also improved significantly (Table 1).

**Table 1:** Changes in patients before and six months after LSG

	Characteristics	Pre-surgery	Post-surgery	*P*
Demographic	BMI (kg/m^2^)	36.75 ± 4.09	26.37 ± 3.47	0.000*
	WC (cm)	115.95 ± 12.41	91.81 ± 11.18	0.000*
	HC (cm)	117.15 ± 8.55	100.90 ± 9.87	0.000*
Glycometabolism	FPG (mmol/L)	5.83 ± 1.55	5.17 ± 0.34	0.022*
	FCP (nmol/L)	3.76 ± 1.09	2.21 ± 0.64	0.000*
	FINS (uIU/mL)	22.36 ± 8.48	9.80 ± 6.40	0.000*
	HbA1c (%)	6.05 ± 0.96	5.05 ± 0.25	0.000*
Liver Function	ALT(IU/L)	63.80 ± 54.32	14.17 ± 7.40	0.000*
	AST(IU/L)	40.00 ± 38.54	15.37 ± 3.86	0.001*
	γ-GGT(IU/L)	45.71 ± 43.68	12.73 ± 6.03	0.000*
	LSM (kPa)	10.03 ± 3.98	6.83 ± 2.95	0.001*
	CAP (dB/m)	330.21 ± 22.73	258.17 ± 27.47	0.000*
Lipid Metabolism	TG (mmol/L)	2.09 ± 1.15	1.11 ± 0.39	0.000*
	TC (mmol/L)	4.91 ± 0.86	4.61 ± 0.85	0.011*
	HDL-C (mmol/L)	1.12 ± 0.23	1.29 ± 0.27	0.000*
	LDL-C (mmol/L)	3.18 ± 0.86	2.80 ± 0.75	0.002*
Inflammation and uric acid	WBC (10^^9^/L)	6.90 ± 1.43	5.66 ± 1.06	0.000*
	Uric Acid (umol/L)	444.31 ± 112.80	355.68 ± 88.06	0.000*

These results suggest that LSG not only reduces weight but also significantly improves glucose and lipid metabolism, fatty liver, uric acid levels, systemic inflammation, and other obesity-related complications in obese patients.

### LSG improves gut microbiota diversity

**Fig. 1:**
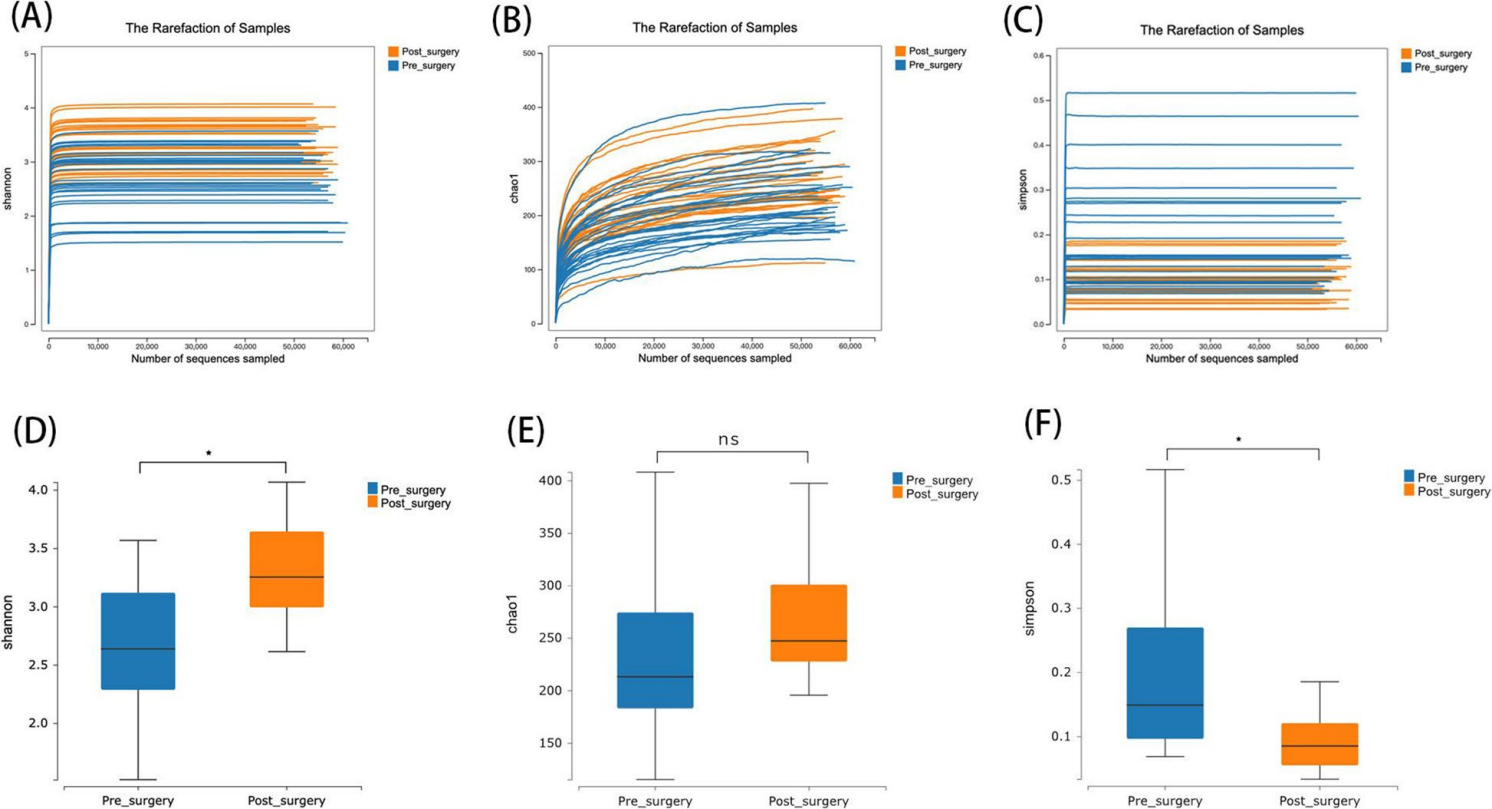
Changes in Gut Microbiota Diversity Before and After Surgery. **A**, **B**, and **C **display rarefaction curves for pre- and post-surgery samples. The x-axis represents the number of sequences sampled, while the y-axis shows different diversity indices: (**A**) Shannon, (**B**) Chao1, and (**C**) Simpson. **D**, **E**, and **F **present box plots comparing pre- and post-surgery diversity indices: (**D**) Shannon diversity, (**E**) Chao1 index, and (**F**) Simpson diversity (*p < 0.05)

We assessed the α-diversity of pre- and post-operative samples using dilution curves, which showed that the sequencing depth was sufficient (Fig. 1A-C). The Shannon curve stabilized at maximum sequencing depth, indicating that the bacterial diversity in the samples was fully detected. Postoperative samples showed an increase in both the Chao1 and Shannon diversity indices, while the Simpson index decreased (Fig. 1D and E). These findings suggest that LSG led to an increase in the total number and richness of gut microbiota species, but a decrease in evenness compared to preoperative samples, indicating that bariatric surgery may be associated with changes in gut microbiota.

**Fig. 2:**
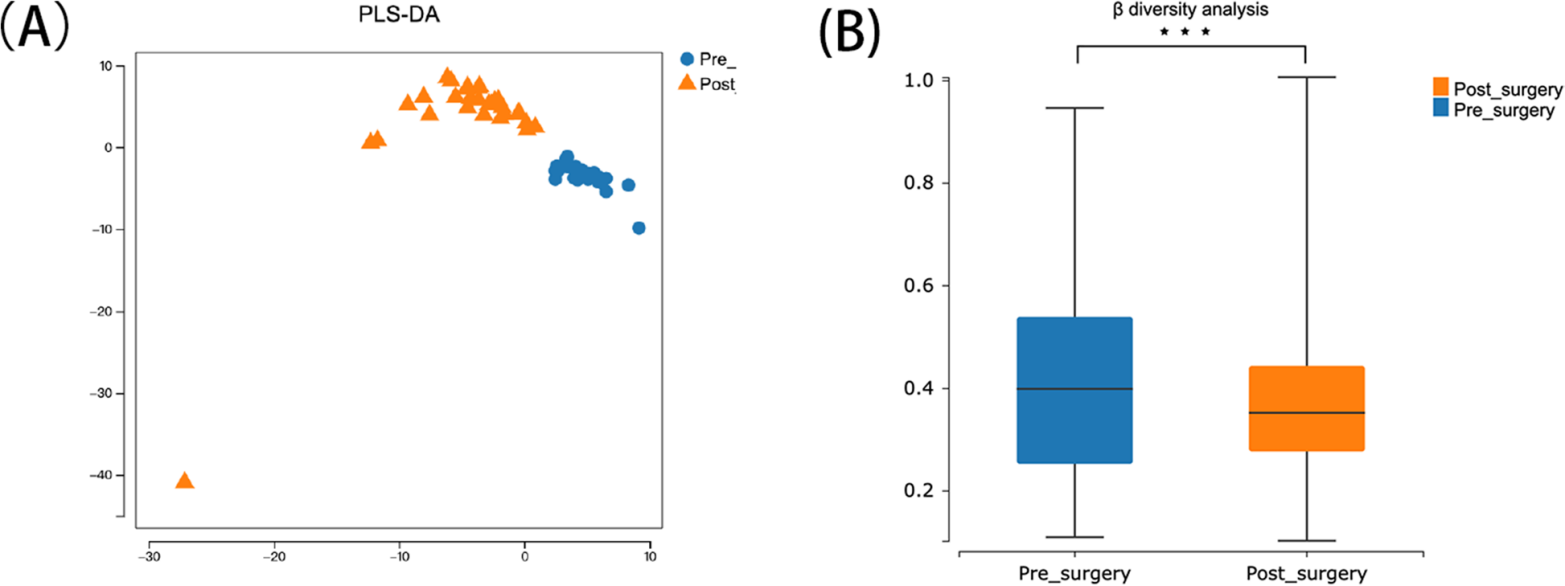
Significant Changes in Gut Microbiota Composition. **A** shows PLS-DA plot illustrating the differences in microbial communities before and after surgery. **B** presents a box plot comparing β-diversity distances between pre-surgery and post-surgery groups

Partial Least Squares Discriminant Analysis (PLS-DA)analysis revealed significant differences in microbial communities between pre- and post-operative samples (Fig. 2A). Additionally, β-diversity analysis showed a decrease in microbial beta diversity following bariatric surgery (Fig. 2B). These findings suggest that bariatric surgery alters gut microbiota composition, highlighting the potential relationship between microbial imbalance and metabolic changes.

### Changes in gut microbiota composition and function after LSG

**Fig. 3:**
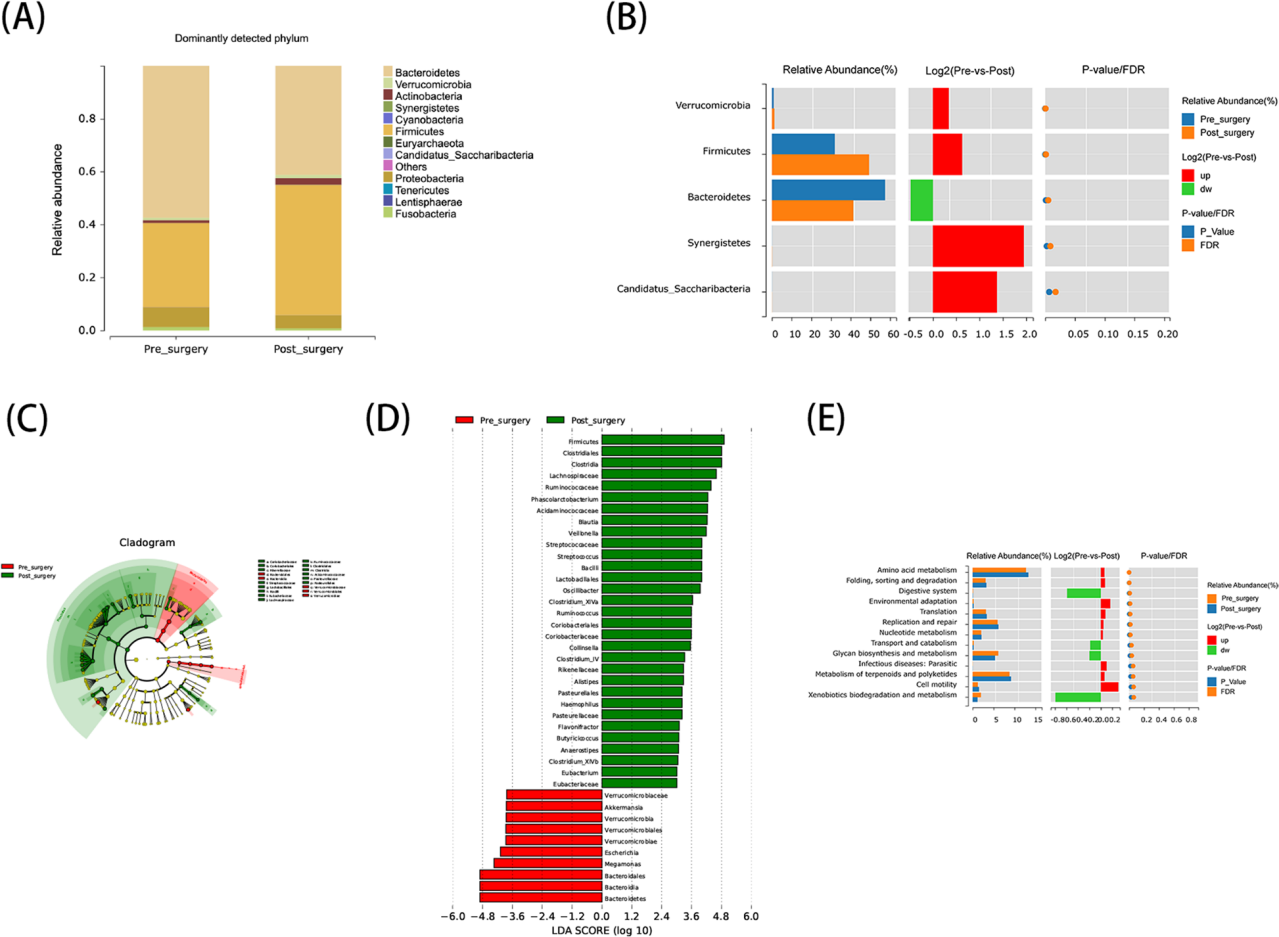
Differential gut microbiota composition before and after surgery. **A** shows the gut microbiota composition at the phylum level. **B** represents the relative abundance of gut microbiota at the phylum level. **C** identifies the taxa that differ significantly between the two groups. **D** provides a phylogenetic tree depicting microbial taxa with significant differences in abundance between pre- and post-surgery groups. **E** represents the functional analysis of microbial metabolic pathways before and after surgery

Species abundance tables at different taxonomic levels were created using OTU annotations to compare species composition in the pre- and post-surgery groups. LSG caused significant changes in the abundance of 5 phyla, 5 classes, 11 orders, 18 families, and 26 genera (FDR, *p* < 0.05, Supplementary Table 1). *Firmicutes* and *Bacteroidetes* were the dominant phyla in both groups (Fig. 3A). Specifically, the preoperative group had a higher proportion of *Bacteroidetes* (57.39% vs. 41.30%) and a lower proportion of *Firmicutes* (49.31% vs. 31.95%) compared to the postoperative group (Fig. 3B). Further analysis at different taxonomic levels revealed significant inter-group differences in 46 genera (FDR, *p* < 0.05). The postoperative group showed increased abundance of *Phascolarctobacterium* (*p* < 0.05), *Blautia* (*p* < 0.001), *Veillonella* (*p* < 0.001), *Streptococcus* (*p* < 0.05), and *Oscillibacter* (*p* < 0.05), while *Fretibacterium* (*p* < 0.05) was less abundant compared to the preoperative group.

LEfSE analysis at the genus level generated a cladogram, while LDA effect size analysis produced a dendrogram to visualize species differences between the two groups, identifying specific microbial communities associated with the postoperative group (Fig. 3C). Setting the LefSe threshold at 3.0 revealed dominant taxa in the postoperative group, including families *Lachnospiraceae*, *Ruminococcaceae*, *Acidaminococcaceae*, and *Coriobacteriaceae*. At the genus level, *Phascolarctobacterium*, *Blautia*, *Veillonella*, and *Streptococcus* were prominent. LDA effect size distribution was used to estimate the impact of each significantly different taxon between groups (Fig. 3D).

Functional analysis of the gut microbiome was performed using the KEGG Pathway database, comparing functional differences between the two groups at Level 3 (Fig. 3E). The results showed that pathways primarily involved metabolism, including amino acid metabolism, glycan biosynthesis, and the metabolism of terpenoids and polyketides. These findings highlight significant shifts in microbial metabolic functions related to amino acid metabolism and glycometabolism after surgery, suggesting that LSG may improve metabolic function through gut microbiota alterations.

### Correlation between differential microbiota and clinical indicators

**Fig. 4:**
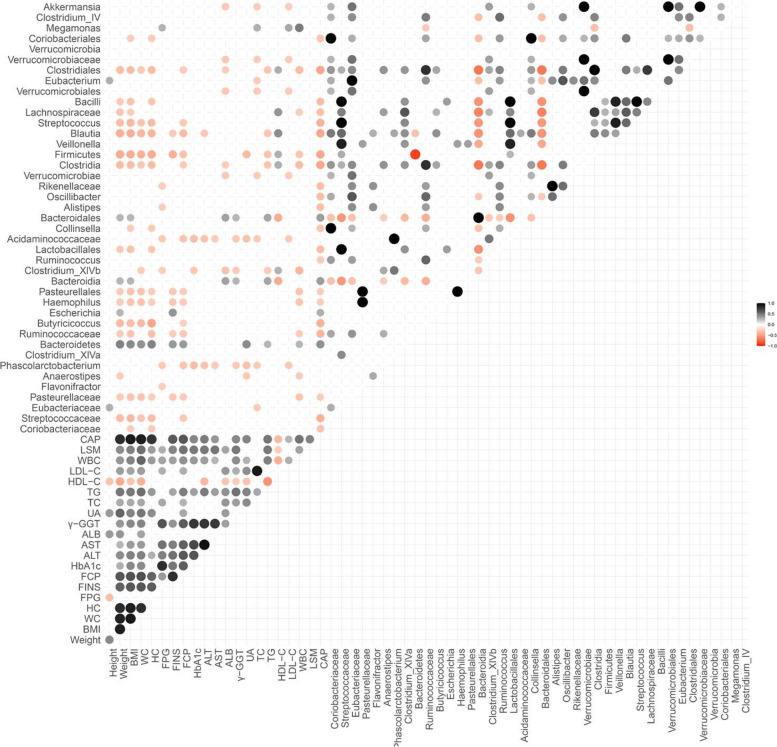
Correlation matrix between gut microbiota and clinical indicators

To assess whether the differential microbiota correlates with clinical indicators, Spearman correlation coefficient analysis was conducted between key clinical indicators and gut microbiota changes (Fig. 4). *Lachnospiraceae* was significantly correlated with patient weight, BMI, HDL-C, and WBC count (*P* < 0.05,Supplementary Table 2). *Ruminococcaceae* showed significant associations with weight, FCP, and FINS levels (*P* < 0.05). *Akkermansia* was negatively correlated with liver stiffness markers such as CAP, LSM, and γ-GGT, suggesting a potential protective role in liver metabolism.

*Lachnospiraceae* and *Ruminococcus* were positively correlated with lipid metabolism markers like LDL-C, HDL-C, and TG, while *Akkermansia* exhibited a weak negative correlation with TG. *Acidaminococcaceae* and *Phascolarctobacterium* demonstrated significant correlations with blood glucose, lipid levels, and liver function. *Blautia* showed notable effects on improving weight, pancreatic function, and lipid metabolism. *Escherichia* and *Firmicutes* displayed strong positive correlations with BMI and liver function markers (ALT, AST, γ-GT), indicating their association with liver dysfunction and metabolic disturbances.

These findings suggest a potential link between specific gut microbes and clinical outcomes, highlighting how changes in gut microbiota after LSG may contribute to improvements in clinical metabolic parameters.

This heatmap illustrates the correlation between different bacterial taxa and various clinical parameters, including metabolic markers (e.g., weight, LDL-C, HDL-C, TG), liver function markers (e.g., ALT, AST), and inflammatory markers (e.g., WBC, CRP). Each circle represents the strength and direction of the correlation: black indicates a positive correlation, while red indicates a negative correlation. The intensity of the color reflects the correlation coefficient, with darker shades representing stronger correlations.

## Disscuss

Globally, the number of obese adults was projected to increase from 400 million in 2005 to over 700 million by 2015, with this trend expected to continue until 2030 [22]. Overweight and obesity pose significant clinical and public health challenges worldwide, and obesity is closely associated with changes in gut microbial diversity, bacterial gene expression, and metabolic pathways. Individuals with reduced gut microbiota diversity are more susceptible to obesity, insulin resistance, and lipid disorders [23]. This study investigated changes in gut microbiota and metabolic indicators in Chinese obese patients after undergoing LSG. Our results show alterations in gut microbiota composition and increased diversity in postoperative samples. LSG led to significant reductions in BMI, improvements in glycemic and lipid metabolism, and notable alleviation of fatty liver severity compared to preoperative conditions.

There are significant population differences in species-level diversity, with *Bacteroidetes* and *Firmicutes* being the dominant phyla in gut microbiota, consistent with previous studies [24]. At the phylum level, the *Firmicutes/Bacteroidetes* ratio was lower in preoperative samples of Chinese obese patients, but six months after LSG, *Bacteroidete*s decreased and *Firmicutes* increased, resulting in a higher *Firmicutes/Bacteroidetes* ratio. This finding contradicts the results of Koliada et al. [15, 25], but aligns with most other studies [17, 26, 27]. Variations in gut microbiota are reasonable, as studies by Deschasaux et al. and He et al. have demonstrated that ethnicity and geographic location significantly influence gut microbiota diversity and composition [28, 29]. Other factors, including diet, lifestyle, geography, and ethnicity, also play a role [30, 31]. Current knowledge about gut microbiota composition related to health is primarily derived from studies conducted in European and North American populations. More research involving diverse ethnic groups and geographical regions is necessary to obtain more comprehensive insights.

Through 16S rRNA analysis, we observed a significant increase in *Verrucomicrobia* after surgery, consistent with the findings of Palmisano et al. [32]. *Akkermansia muciniphila*, a representative bacterium of *Verrucomicrobia*, is negatively correlated with obesity, diabetes, cardiovascular diseases, and low-grade inflammation, making it a potential candidate for treating type 2 diabetes and obesity [33, 34]. The postoperative changes in gut microbiota are linked to improvements in metabolic status and inflammation, suggesting that alterations in microbiota due to weight loss surgery may contribute to weight reduction and improvements in obesity-related comorbidities, as observed in our LEfSe analysis [35].

At the genus level, *Lachnospiraceae*, *Ruminococcaceae*, *Acidaminococcaceae*, and *Coriobacteriaceae* were predominant in the postoperative gut microbiota. Studies have shown that decreased levels of short-chain fatty acid (SCFA)-producing bacteria, such as those from these groups, are associated with conditions like inflammatory bowel disease (IBD), cancer, obesity, and diabetes [36]. These bacterial families are significant producers of SCFAs like butyrate and propionate, which enhance the immune system, regulate lipid metabolism, and help prevent tumor development [37]. SCFAs also stimulate peptide secretion via G protein-coupled receptors on gut L cells, promoting the secretion of GLP-1 and PYY, which contribute to weight loss and improved insulin resistance [38–41].*Lachnospiraceae is a* major family in the healthy adult gut, comprising 10–45% of the total bacterial population in feces [42]. It has been shown to reduce inflammation through SCFA and secondary bile acid production, lowering the incidence of IBD, including Crohn's disease [43, 44].

The composition of gut microbiota is closely linked to human lifespan. Research in *The Lancet* indicates that metabolic surgery significantly reduces mortality and extends life expectancy compared to conventional obesity management in adults [45]. In addition, increased *Ruminococcaceae* abundance has been observed in long-lived populations in China [46]. Studies have shown that *Ruminococcaceae* levels increase with age, with the elderly and long-lived individuals (> 90 years old) having significantly higher levels than younger populations [47].Postoperative patients showed significant improvements in blood lipid levels, and *Coriobacteriaceae* has been found to alleviate inflammation and improve bile acid and cholesterol metabolism in high-fat diet conditions [48]. *Acidaminococcaceae* has protective effects on the cardiovascular system [49], and *Coriobacteriaceae* are associated with 15 metabolites that may play a role in disease prevention and health improvement [50, 51]. Collectively, *Coriobacteriaceae* contribute to improving lipid metabolism, extending lifespan, regulating blood sugar, and enhancing overall health outcomes [52].

At the family level, *Phascolarctobacterium*, *Blautia*, *Veillonella*, and *Streptococcus* are the dominant taxa. *Phascolarctobacterium* has been significantly reduced in patients with type 2 diabetes mellitus (T2DM), with its abundance negatively correlated with body fat percentage and positively correlated with insulin sensitivity [53, 54]. Research suggests that *Phascolarctobacterium* may improve obesity, insulin sensitivity, adipose tissue inflammation, and atherosclerosis by beneficially altering gut microbiota composition [55]. *Blautia* is the only gut microbe significantly negatively correlated with visceral adipose tissue [56]. It has been identified as a key gut microbiota associated with weight loss in middle-aged obese Korean women [57]. In diabetic children, *Blaut*ia abundance is significantly reduced compared to healthy children [58]. Studies indicate that *Blautia* can inhibit insulin signaling and fat accumulation in adipocytes by activating G protein-coupled receptors GPR41 and GPR43, promoting lipid and glucose metabolism in other tissues, and alleviating obesity-related complications, particularly those associated with inflammation [59, 60].

Both weight loss surgery and changes in dietary habits lead to significant alterations in gut microbiota. In our study, we observed the proliferation of beneficial bacteria and the reduction of potential pathogens. Long-term follow-up studies of patients who undergo weight loss surgery suggest that surgery influences the microbial community by selecting for beneficial bacteria, potentially outweighing any risks posed by pathogens.

This is the first study in central China investigating changes in gut microbiota composition in obese adults undergoing LSG. However, our sample size is small, and the follow-up period is short. As this is an observational study, further functional studies are needed to fully understand the role of gut microbiota in weight loss and the metabolic improvements observed after LSG. Continued research into the relationship between gut microbiota and the host, as well as its impact on human health, may contribute to the development of microbiota-based therapies for metabolic disorders.

## Conclusion

The weight loss and metabolic improvements observed following LSG result from complex mechanisms involving hormonal, anatomical, and gut microbiota modifications. In addition to significantly reducing body weight, LSG may alleviate metabolic syndrome and other comorbidities by altering gut microbiota.

## Supplementary Information

Supplementary Material 1.

## Data Availability

The datasets used and/or analysed during the current study are available from the corresponding author on reasonable request.

## Abbreviations

LSG: Laparoscopic sleeve gastrectomy
BMI: Body mass index
RCT: Randomized clinical trial
FPG: Fasting plasma glucose
HbA1c: Hemoglobin A1C
TC: Total cholesterol
LDL-C: Low-density lipoprotein cholesterol
HDL-C: High-density lipoprotein cholesterol
TG: Triglycerides
ALT: Alanine aminotransferase
AST: Aspartate aminotransferase
UA: UAric acid
LEfSe: Linear discriminant analysis effect size
LDA: Linear Discriminant Analysis
WC: Waist circumference
HC: Hip circumference
FCP: Fasting plasma C-peptide
FINS: Fasting serum insulin
γ-GGT: γ-Glutamyl Transferase
WBC: White blood cell
LSM: Liver stiffness measurement
CAP: Controlled attenuation parameter
PLS-DA: Partial Least Squares Discrimination Analysis
SCFAs: Short-chain fatty acids
IBD: Inflammatory bowel disease
